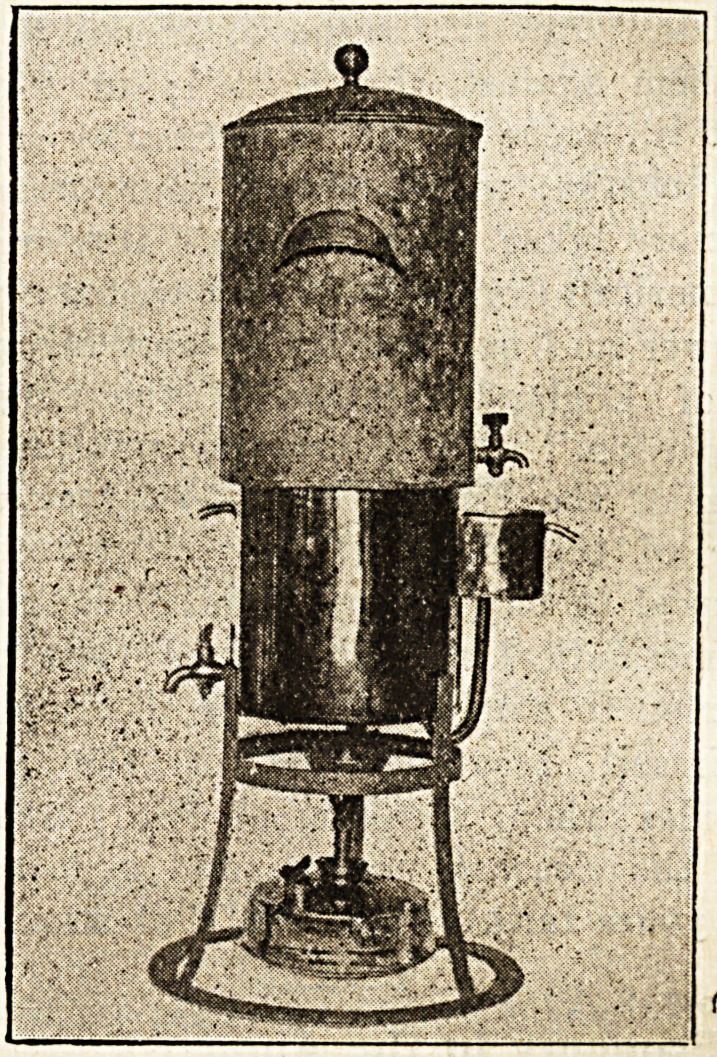# Institutional Needs

**Published:** 1916-06-17

**Authors:** 


### Institutional Needs.

### THE "WARDEN" HOSPITAL WATER STERILISER.

LAWRENCE PATENT WATER SOFTENER AND STERILISER CO., LTD., Parliament Mansions, Victoria Street, S.W.

STERILISED supplies of both hot and cold water are such an indispensable feature of the modern operating theatre, as well as of other departments of a hospital, that there is ample scope for simple yet efficient machinery for ensuring them at a minimum of expense and trouble. The firm which manufactures the "Warden" patterns has evidently studied the problems involved from the point of view of varying types of institution, for their products are supplied in many different sizes as well as in different designs. Pattern "A" of the series comprises a sterilising chamber designed especially for the *rapid* heating of cold water, and so arranged that the water cannot pass the outlet tap until it has reached a temperature sufficient to sterilise it. The reservoir of cold water has to be filled by hand. Pattern "B" is designed for *automatic* use where there is a water supply tank. Then there is a combined hot and cold water steriliser, from which a supply of either hot or cold sterilised water can be obtained at will. The special features of these equipments upon which stress is laid are their simplicity—no safety valve, thermometer, or water gauge is necessary; their automatic action; the certainty of full sterilisation; the fact that to ensure cold sterile water as well as hot there is no need of a separate cold-water supply; and, finally, their economical working.

## Figures and Tables

**Figure f1:**